# Combinatorial Regulation of Meiotic Holliday Junction Resolution in *C. elegans* by HIM-6 (BLM) Helicase, SLX-4, and the SLX-1, MUS-81 and XPF-1 Nucleases

**DOI:** 10.1371/journal.pgen.1003591

**Published:** 2013-07-18

**Authors:** Ana Agostinho, Bettina Meier, Remi Sonneville, Marlène Jagut, Alexander Woglar, Julian Blow, Verena Jantsch, Anton Gartner

**Affiliations:** 1 Centre for Gene Regulation and Expression, University of Dundee, Dundee, United Kingdom; 2 Department of Chromosome Biology, Max F. Perutz Laboratories, University of Vienna, Vienna, Austria; National Cancer Institute, United States of America

## Abstract

Holliday junctions (HJs) are cruciform DNA structures that are created during recombination events. It is a matter of considerable importance to determine the resolvase(s) that promote resolution of these structures. We previously reported that *C. elegans* GEN-1 is a symmetrically cleaving HJ resolving enzyme required for recombinational repair, but we could not find an overt role in meiotic recombination. Here we identify *C. elegans* proteins involved in resolving meiotic HJs. We found no evidence for a redundant meiotic function of GEN-1. In contrast, we discovered two redundant HJ resolution pathways likely coordinated by the SLX-4 scaffold protein and also involving the HIM-6/BLM helicase. SLX-4 associates with the SLX-1, MUS-81 and XPF-1 nucleases and has been implicated in meiotic recombination in *C. elegans*. We found that *C. elegans [mus-81; xpf-1], [slx-1; xpf-1]*, *[mus-81; him-6]* and *[slx-1; him-6]* double mutants showed a similar reduction in survival rates as *slx-4*. Analysis of meiotic diakinesis chromosomes revealed a distinct phenotype in these double mutants. Instead of wild-type bivalent chromosomes, pairs of “univalents” linked by chromatin bridges occur. These linkages depend on the conserved meiosis-specific transesterase SPO-11 and can be restored by ionizing radiation, suggesting that they represent unresolved meiotic HJs. This suggests the existence of two major resolvase activities, one provided by XPF-1 and HIM-6, the other by SLX-1 and MUS-81. In all double mutants crossover (CO) recombination is reduced but not abolished, indicative of further redundancy in meiotic HJ resolution. Real time imaging revealed extensive chromatin bridges during the first meiotic division that appear to be eventually resolved in meiosis II, suggesting back-up resolution activities acting at or after anaphase I. We also show that in HJ resolution mutants, the restructuring of chromosome arms distal and proximal to the CO still occurs, suggesting that CO initiation but not resolution is likely to be required for this process.

## Author Summary

Meiosis is a specialized cell division, where a single S-phase is followed by two rounds of cell divisions to ensure the production of haploid gametes. Meiotic crossover (CO) recombination is required for genetic diversity and for the proper segregation of chromosomes during the first meiotic division. In addition, in organisms with holocentric chromosomes COs are required for differentiating meiotic chromosomes into distinctly organized CO distal and CO proximal domains. Holliday junctions (HJs) are crucial intermediates during meiotic recombination, but we still know very little about how they are processed to ensure CO recombination, especially in animals. In this study we analyse the combined requirement of *C. elegans* nucleases and the conserved HIM-6/BLM helicase to regulate the processing of meiotic recombination intermediates. Based on genetic and cytological analysis we propose that HIM-6/BLM and the XPF-1 nuclease, as well as the SLX-1 and MUS-81 nucleases act in two redundant pathways to promote HJ resolution. Furthermore, we provide evidence that CO initiation but not CO resolution is likely important for the differentiation of meiotic chromosomes into CO distal and CO proximal domains.

## Introduction

Homologous recombination is important for error-free DNA double-strand break (DSB) repair and for meiotic crossover (CO) formation. Meiosis is a specialized series of two sequential cell divisions that ensures the reduction of the diploid genome and results in the production of haploid gametes. During meiosis COs generate genetic diversity. Moreover, CO products, which at the level of chromosomes become visible as chiasmata, provide stable connections between maternal and paternal homologous chromosomes (homologues). The connection provided by chiasmata is required for accurate homologue segregation in the first meiotic division.

Meiotic recombination is initiated by the introduction of programmed DSBs [1] by the conserved meiosis-specific Spo11 protein [2]. These DSBs are resected to produce 3′ single-stranded DNA overhangs that, aided by RecA like recombinases (RAD-51 in *C. elegans*), initiate strand invasion into a homologous donor sequence [3]. In most organisms studied so far, the estimated number of induced DSBs during meiosis exceeds the number of COs generated, with ratios as variable as 2∶1 in *S. cerevisiae* up to 20∶1 in maize [3]–[9]. In *C. elegans* only one DSB per homologue pair will result in a CO event [10], [11]. Following strand invasion by the 3′ single-stranded overhang the first recombination intermediate (RI) is referred to as ‘D-loop’ (for review [12], [13]). Helicase-driven D-loop disassembly can occur, which in budding yeast is driven by the Sgs1/BLM-like helicase [14]. Such activities are also ascribed to BLM in animals and further helicases such as RTEL are likely to play a similar role [15]. After D-loop disassembly, the invading 3′ single strand, which has been extended by DNA synthesis, can capture the other broken DNA end and synthesis-dependent strand annealing (SDSA) occurs. SDSA occurs relatively early during meiosis and appears to be set up independently of later RIs which can result in COs, at least in yeast [16]–[19]. Interestingly, in *C. elegans* deletion of the RTEL helicase, which can promote D-loop disassembly in vitro, leads to an elevated number of meiotic COs [15], [20]. In contrast, deletion of *him-6*, the *C. elegans* BLM homologue, results in reduced meiotic CO formation consistent with the occurrence of an increased number of unconnected homologues visible as univalents in oocytes of strong *him-6* mutants [21].

When the D-loop remains intact, second DNA end capture by the extended invading single-strand leads to a cruciform DNA structure called Holliday junction (HJ). Such RI was originally postulated in 1964 [22] and a refined model predicted that the majority of HJs occur as double HJs (dHJs) [23]. Direct evidence for the occurrence of dHJs as RIs during meiosis (and during DSB repair in diploid mitotic cells [24]) was obtained in budding yeast [25], [26], while in fission yeast single HJs appear to be predominant [25]. dHJs can be processed in various ways and result in either a CO or a non-CO (NCO). In a process referred to as dHJ dissolution, coupled helicase and topoisomerase activities conferred by Sgs1/BLM and Top3-Rmi1 can disassemble dHJs, resulting in a NCO [27]. Alternatively, dHJs can be resolved by nucleases (for review see [28], [29]). Depending on the symmetry of the cleavage, either COs or NCOs arise.

Canonical HJ resolvases, such as RuvC and RusA, were first described in bacteria and bacteriophages [30]–[32]. These resolvases confer symmetrical cleavage of HJ substrates so that cleavage products can be re-ligated in vitro. In other organisms, nuclear ‘canonical’ resolvases remained elusive [33]. The first such purified activity was shown to be conferred by an N-terminal fragment of the human Gen1 nuclease albeit a lower level activity towards FLAP structures is detectable [34]. The respective budding yeast (Yen1) and *C. elegans* proteins (GEN-1) also confer in vitro HJ resolution [34], [35]. In budding yeast *yen1* single mutants do not show an obvious recombinational repair or meiosis defect [36]–[38]. Gen1 is absent in fission yeast. In *C. elegans gen-1* mutants are defective in recombinational repair and DNA damage checkpoint signalling while no overt meiotic phenotype is apparent [35]. There is emerging evidence that HJ resolution might not necessarily be conferred by symmetrically cleaving ‘canonical’ resolvases. Rather (combinations of) non-symmetrically cleaving nucleases as well as helicases might confer the resolution of HJs. A dominant role of the Mus81 nuclease and its regulatory subunit Eme1/Mms4 in meiotic HJ resolution is evident in fission yeast, and the associated meiotic defect can be bypassed by expressing bacterial RusA resolvase [39]. Mus81 catalyses multiple structure-specific nuclease reactions: it cleaves FLAP structures and D-loops and has a very high affinity for nicked HJs in vitro. Cleavage of intact HJs by Mus81 in vitro occurs with low activity (for review [29]). In budding yeast *mus81 yen1* double mutants meiotic chromosome segregation is perturbed due to persistent chromatin linkages [40]. Yen1 is kept inactive in the first meiotic cell division by phosphorylation, but becomes active in the second meiotic division, where it appears to provide a back-up function if chromosomes remain entangled after the first meiotic division [40]. Nevertheless, the rate of meiotic recombination is only slightly reduced in *mus81 yen1* double mutants [40]. MUS81 deletion confers only ‘minor’ meiotic phenotypes in mice [41] and in *C. elegans* MUS-81's activity only becomes apparent in mutants producing aberrant CO products [20], [42].

Another possible factor contributing to HJ resolution is the conserved Slx4 scaffold protein with its associated nucleases Xpf1, Mus81 and Slx1 [42]–[48]. Xpf1 is a FLAP endonuclease that together with Ercc1 is required for the 5′ incision in nucleotide excision repair [49], as well as for cleaving FLAP structures in the single-strand annealing DNA repair pathway [50], [51]. In the fruit fly *mei-9/xpf1* mutants have dramatically reduced rates of meiotic recombination [52], and this was also seen in *mus312/slx4*, albeit with even lower meiotic recombination levels [43]. Genetic evidence suggests that MUS312/Slx4 and MEI-9/Xpf1 might act as a complex to exert this function in the fly [43]. A reduced recombination rate together with occasional DNA threads linking meiotic chromosomes were also reported for *C. elegans slx-4* (also termed *him-18*) [42]. An overt role of Xpf1 in meiotic HJ resolution has not been documented for any other organism. Recent evidence suggests that *C. elegans* SLX-1 does not affect the frequency but the distribution of meiotic CO, such that recombination events are shifted toward the centre of chromosomes in *slx-1* mutants [47].

In budding yeast an *sgs1*, *mms4 (mus81)*, *yen1*, *slx1* quadruple mutant exhibits a severe reduction but not complete elimination of meiotic COs. It was also reported that the Exo1 nuclease and the mismatch-repair proteins Mlh1 and Mlh3 contribute to HJ resolution [53]. Accordingly, no COs were observed in yeast *mlh3*, *sgs1*, *mms4 (mus81)*, *yen1*, *slx1* quintuple mutants, when RIs and CO products were assessed employing a recombination hotspot system [14], [54]. It remains to be shown how the interplay of these nucleases and the Sgs1 helicase contributes to CO formation. In addition very little is known about meiotic HJ resolution in animals.

Here we report a systematic analysis of the role of various nucleases in promoting the resolution of meiotic RIs, likely HJs, in *C. elegans*. We found no meiotic phenotypes associated with *gen-1* neither as a single mutant nor in combination with *him-6* or various nuclease mutants. In contrast, *[mus-81; xpf-1]*, *[slx-1; xpf-1]*, *[mus-81; him-6]* and *[slx-1; him-6]* but not *[xpf-1; him-6]* or *[mus-81 slx-1]* double mutants show synthetic meiotic phenotypes consistent with a defect in resolving/processing meiotic RIs. A large proportion of chromosomes fail to form bivalents, instead univalent structures linked by distinct chromatin bridges become apparent using high-resolution microscopy. Our data also indicate that XPF-1, MUS-81 and SLX-1 might exert their meiotic function in complex with the SLX-4 scaffold protein. Our results are consistent with the existence of at least two redundant meiotic resolvase activities: one requiring HIM-6 and XPF-1, and the other dependent on SLX-1 and MUS-81. Furthermore, we found that CO designation as well as the subsequent differentiation of CO distal and proximal chromosome arms still occurs when HJ resolution is impaired and these events are therefore likely determined by an earlier step of CO maturation.

## Results

### XPF-1-HIM-6 and SLX-1-MUS-81 appear to act in two redundant pathways both of which require SLX-4

**Figure 1:**
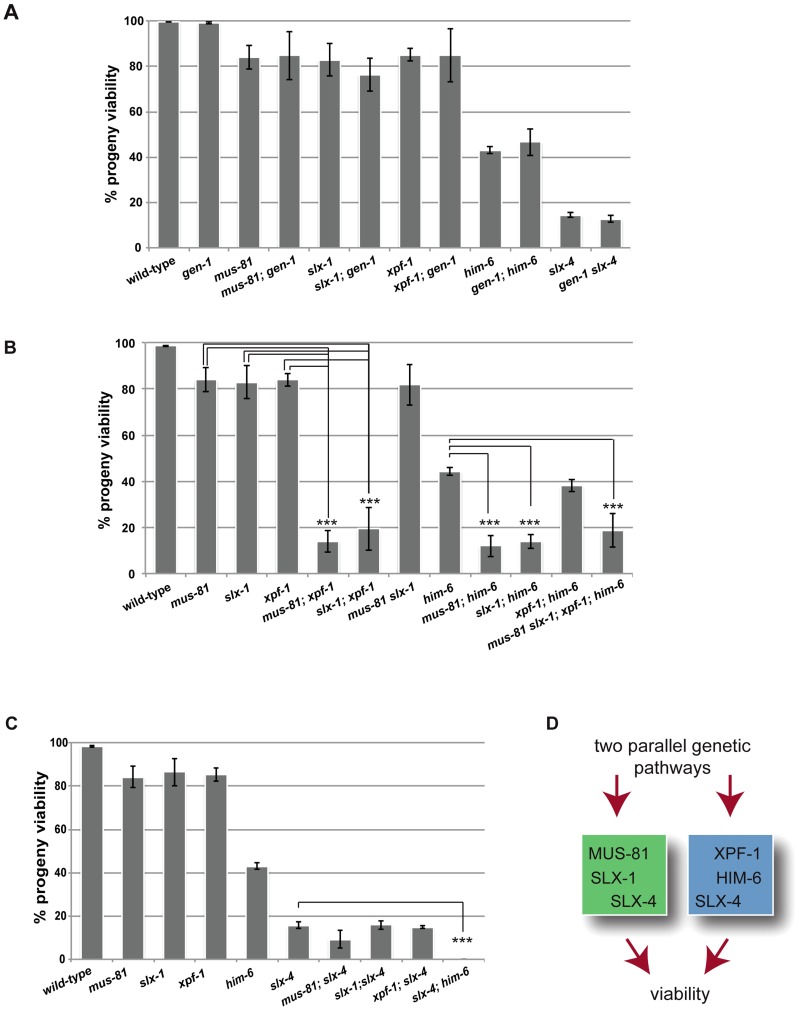
Dissection of genetic interactions between *gen-1, mus-81, slx-1, xpf-1*, *him-6 and slx-4*. (A) *gen-1* does not alter viability, alone or in conjunction with nucleases, *slx-4* or *him-6*. Progeny viability in % was determined as described in Material and Methods. (B) Evidence for MUS-81/SLX-1 and XPF-1/HIM-6 acting in two genetic pathways. Scoring was done as in A. For double mutants with reduced viability the F1 progeny of double homozygous mothers derived from hT2-balanced lines was scored (for strain list see Materials and Methods). In contrast *slx-1; him-6* double mutants derived from a strain were *him-6* is balanced by nT1 behave as synthetic lethal (data not shown) [47]. We think that this lethality is due the nT1 balancer, which has been described as prone to brake down, as preferably segregating to the male germ line and as conferring a reduced brood size [88]. (C) Reduced progeny viability of *slx-4* is not enhanced by nuclease mutations. (D) Genetic model. Asterisks indicate statistical significance between different genotypes as determined by two-tailed Mann-Whitney test (*** indicates P<0.01).

Given the absence of an overt meiotic phenotype of YEN1/GEN1 in budding yeast and *C. elegans* we wished to investigate if *C. elegans* GEN-1 might act redundantly with other nucleases in meiosis. A reduction in viability and a high incidence of male (*him*) phenotype, associated with X-chromosome non-disjunction, can be indicative of a meiotic defect. While the progeny hatch-rate of *gen-1* (99.1%±0.4%) was similar to wild-type (99.7%±0.2), a slight reduction was observed for *xpf-1* (85%±2.7%), *mus-81* (84%±5.1%), and *slx-1* (83%±7.2%) single mutants (Figure 1A). *him-6* and *slx-4* mutants showed 43%±1.7% and 14.5%±1.2% viability respectively, in accordance with previous reports (Figure 1A) [21], [42]. Combining *gen-1* with any of these mutants, that were extensively outcrossed to N2 wild-type, did not result in a significant reduction in progeny viability (Figure 1A). In contrast, viability was significantly reduced to ∼15–20% in *[mus-81; xpf-1]*, *[slx-1; xpf-1]* as well as in *[mus-81; him-6]* and *[slx-1; him-6]* double mutants (P<0.01 in all cases) (Figure 1B). These double mutants as well as any other compound mutants with reduced viability were maintained as balanced lines and phenotypes were analysed in the first homozygous generation (see Materials and Methods). Double mutants of *slx-1* or *mus-81* with a second *him-6* allele resulted in a similar phenotype, as did *[mus-81 ercc-1]* or *[slx-1 ercc-1]* double mutants (data not shown). Ercc1 forms a heterodimer with Xpf1 from yeast to human [55]. In contrast, progeny survival of *[mus-81 slx-1]* and *[xpf-1; him-6]* was not reduced compared to the respective single mutants (Figure 1B). Analysing a *[mus-81 slx-1; xpf-1; him-6]* quadruple mutant revealed a reduction in viability similar to that observed in the double mutants (Figure 1B). In summary, our genetic data indicate that these genes might act in two redundant pathways for viability: one requiring XPF-1 and HIM-6 the other requiring SLX-1 and MUS-81. We also note that the reduction of viability observed in *[mus-81; xpf-1]*, *[slx-1; xpf-1]*, *[mus-81; him-6]* and *[slx-1; him-6]* double mutants equals the reduced viability observed in *slx-4* mutants (Figure 1A, 1B). In addition, the reduced viability of *slx-4* is not enhanced when *slx-4* is combined with *mus-81*, *slx-1* or *xpf-1* (Figure 1C). As previously reported *[slx-4; him-6]* double mutants showed 0% survival [42], a rate which contrasts with the ∼15–20% hatch rate observed in the aforementioned double mutants and the *[mus-81 slx-1; xpf-1; him-6]* quadruple mutant (Figure 1B, 1C). In summary, these results suggest that the XPF-1-HIM-6 pathway as well as the SLX-1-MUS-81 pathway both require the function of SLX-4 (Figure 1D). This finding is consistent with recent observations identifying Slx4 as a scaffold protein for Mus81, Slx1 and Xpf1 in *C. elegans* and mammalian cells [42]–[48].

The finding that the *[slx-4 him-6]* double mutant is 100% inviable, while the *[mus-81 slx-1; xpf-1; him-6]* quadruple mutant shows 15–20% viability indicates that SLX-4 has additional functions to those conferred by its interaction partners SLX-1, MUS-81 and XPF-1 (Figure 1D).

### XPF-1-HIM-6 and SLX-1-MUS-81 are not required for meiotic chromosome pairing, axis formation and synapsis

**Figure 2:**
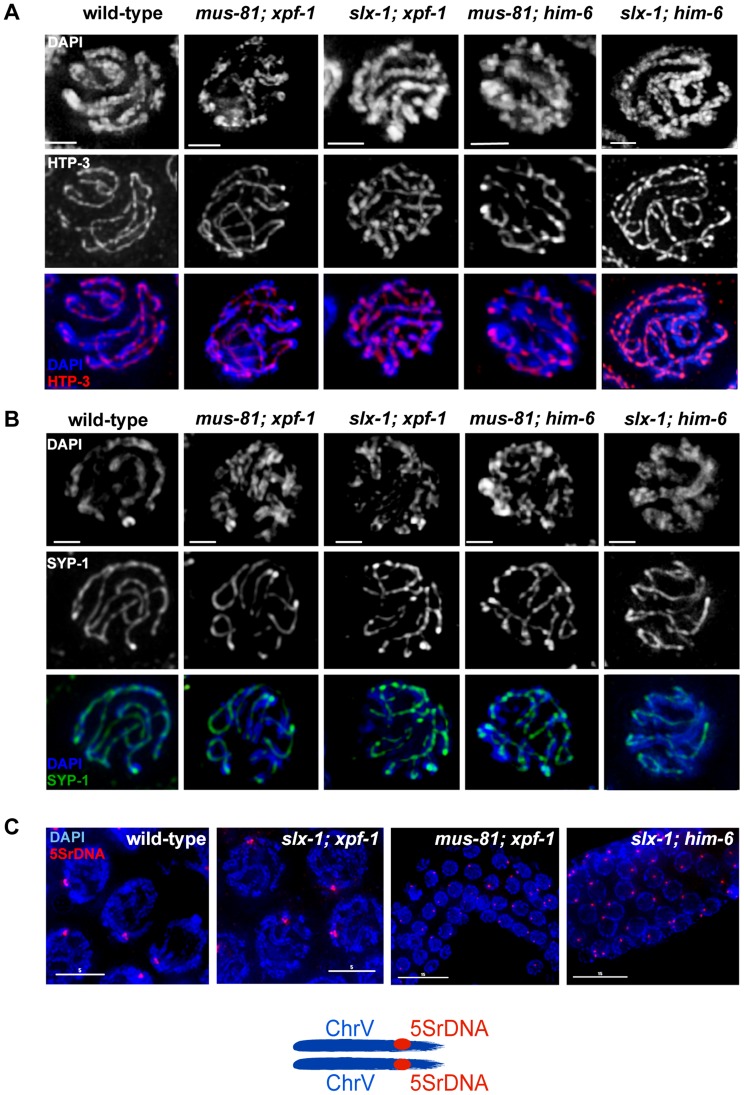
Normal chromosome morphology and HTP-3 and SYP-1 loading in pachytene stage nuclei in *[mus-81; xpf-1], [slx-1; xpf-1], [mus-81; him-6]* and *[slx-1; him-6]* double mutants. (A) Projections of representative nuclei from whole-mount gonads stained with α-HTP-3 antibody (white) and DAPI (white), or red and blue, respectively, in merged images. (B) Representative pachytene nuclei stained with α-SYP-1 antibody and DAPI (SPY-1, green in merged images). Scale bars are shown in white (1 µm). (C) FISH images were taken with 100× and 60× magnification.

Errors in meiotic chromosome segregation can originate from defects in early meiotic events, such as chromosome axis establishment, homologous chromosome pairing and synapsis. Such events could be defective in our double mutants, accounting for the loss of viability. In order to investigate axis morphogenesis, we analysed the localization of HTP-3, a component of the *C. elegans* axial element [56]. Axial elements coordinate homologous pairing and synapsis, as well as homologous recombination. We found that overall chromosome morphology, as well as HTP-3 localization occurred normally during both pachytene and diplotene in all double mutants analysed. HTP-3 was found along the length of parallel DAPI tracks in pachytene (Figure 2A) and associated with chromatin in diplotene (data not shown), a stage in which homologues start to desynapse. To analyse synapsis we immuno-stained for SYP-1, a component of the synaptonemal complex (SC) central region [57]. As is the case for HTP-3, SYP-1 localization in all double mutants was indistinguishable from wild-type during pachytene (Figure 2B). To address chromosome pairing, we used FISH probes to detect the 5S ribosomal DNA locus on chromosome V. Two immuno-fluorescence signals were detected in close proximity to each other, colocalizing with the parallel DAPI-stained tracks on each pachytene nucleus, suggesting that homologue chromosome pairing is unperturbed in all double mutants analysed (Figure 2C). Thus, it appears that the reduced viability observed in *[mus-81; xpf-1]*, *[slx-1; xpf-1]* as well as in *[mus-81; him-6]* and *[slx-1; him-6]* double mutants is not due to a defect in homologous pairing, chromosome axes or synaptonemal complex establishment.

### Abrogation of XPF-1-HIM-6 and SLX-1-MUS-81 leads to chromatin bridges between meiotic chromosomes

**Figure 3:**
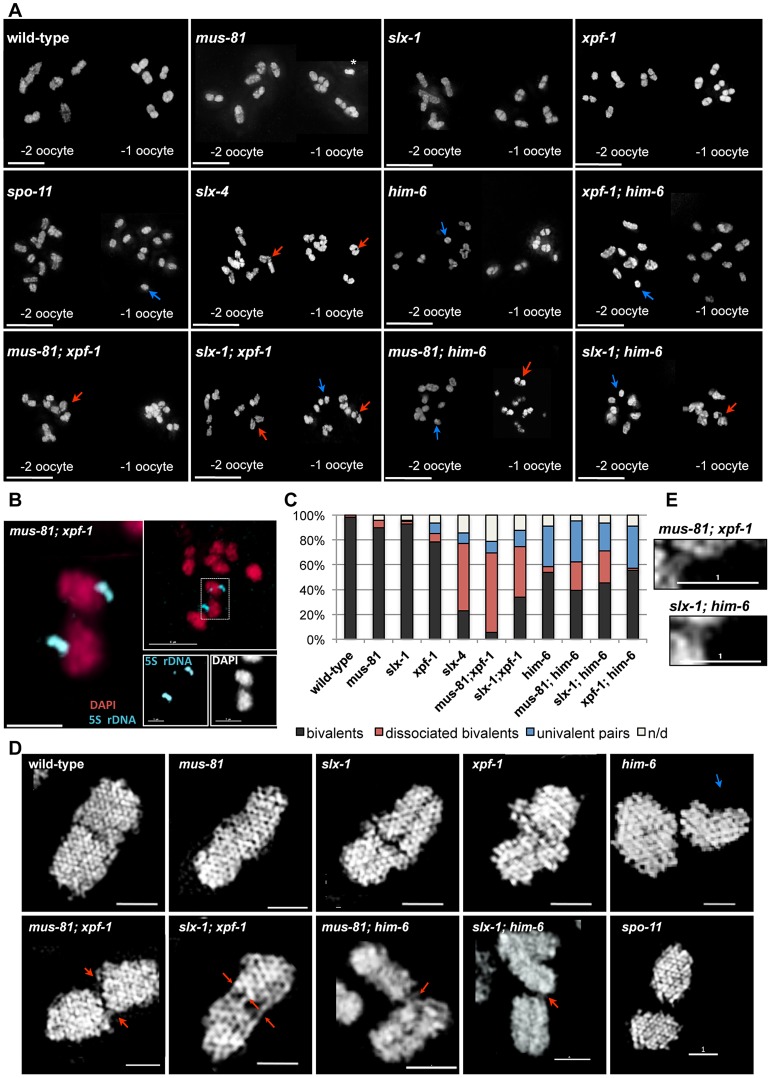
Evidence for DNA bridges linking homologous chromosomes in in *[mus-81; xpf-1], [slx-1; xpf-1], [mus-81; him-6]* and *[slx-1; him-6]* double mutants. (A) DAPI stained diakinesis chromosomes. Images represent projected Z-stacks obtained by deconvolution microscopy. Red arrows indicate thin chromatin bridges, blue arrows indicate univalents. The asterisk points to a sperm nucleus. Scale bars are shown in white (5 µm). (B) FISH analysis reveals that chromatin linkages occur between homologous chromosomes. Scale bars shown in white (2 µm). (C) Quantification of bivalents, ‘dissociated bivalents’ and univalents. 100% would reflect all oocytes scored containing six bivalents. A pair of ‘dissociated bivalents’ as well as two (unlinked) univalents are scored as one, in the respective categories. A minimum of 15 oocytes was scored for each genotype. Chromosomes often overlap in cytological preparations. In these cases chromosomes were scored as “n/d”. In addition, in *mus-81, slx-1* and *[xpf-1; him-6]* in a very limited number of cases, fusions, which appear to occur between different chromosomes could be observed (Figure S1, data not shown, also see co-submitted paper by Saito *et al.* [48]). (D) Images of representative chromosomes taken by super-resolution SIM microscopy. DNA bridges are indicated by red arrows. Scale bars are shown in white (1 µm). (E) Close up of DNA bridges obtained by SIM microscopy.

**Figure 4:**
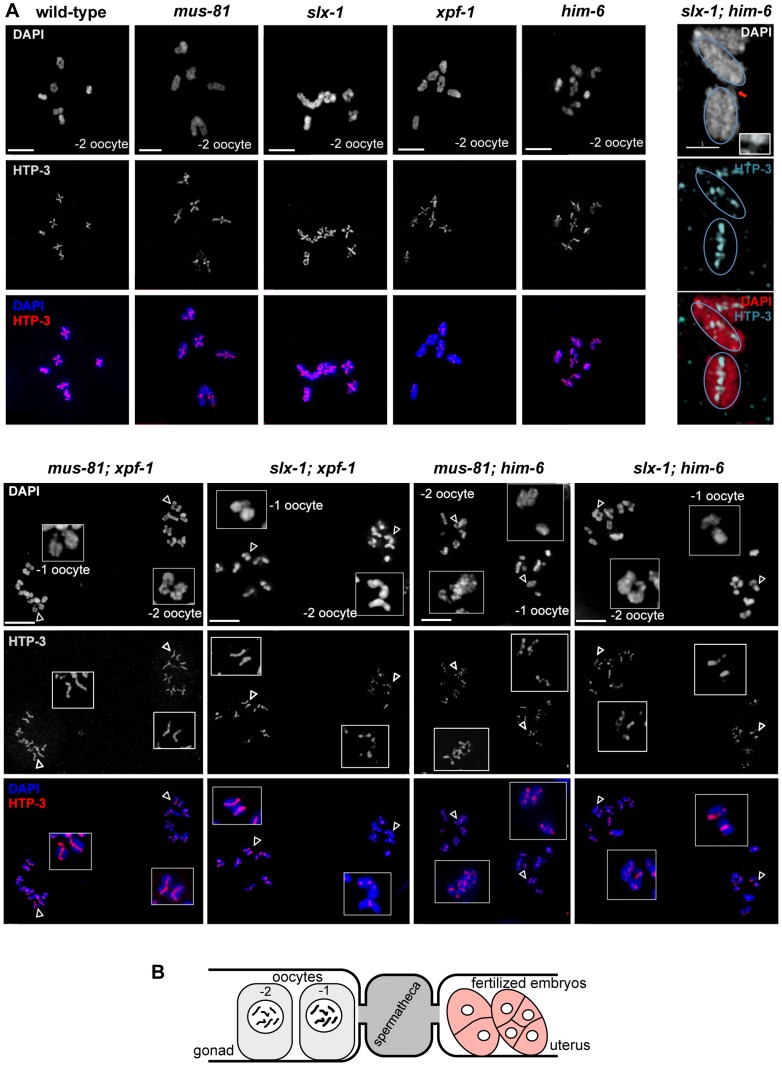
Absence of chiasmata on ‘dissociated bivalents’ revealed by HTP-3 staining. (A) Projections of representative nuclei of diakinesis oocytes stained with α-HTP-3 antibody and DAPI. Open arrowheads indicate the linked chromosomes shown in close-ups. The upper right panels show SIM images of a representative ‘dissociated bivalents’ of an *[slx-1; him-6]* oocyte. Linked univalent are encircled in blue. Scale bars are shown in white (4 µm). (B) Schematic representation of the proximal germ line, spermatheca and early embryogenesis in the *C. elegans* germ line.

To assess if the synthetic phenotypes we observed were due to a defect in DNA repair processes, we analysed meiotic chromosomes in diakinesis. The morphology and number of diakinesis chromosomes can serve as a readout of meiotic recombination defects [58]. During diakinesis, homologous chromosomes pair and restructure forming bivalents. These bivalents can be observed as six DAPI-stained bodies in wild-type maturing oocytes. Defects in meiotic recombination can result in a failure to stably connect homologous chromosomes, which becomes apparent as univalents at diakinesis (12 when physical linkages between all six homologue pairs fail to form). In wild-type we observed six bivalents in the last two oocytes (−1 and −2) prior to fertilisation (Figure 3A, Figure 4B, Video S1). In contrast 12 univalents were apparent in *spo-11* mutants (Figure 3A, Video S7). Consistent with the previously described *him* (high incidence of males through X chromosome non-disjunction) phenotype for *xpf-1* and *him-6* [21], [42], univalents and bivalents were observed in these mutants (Figure 3A, blue arrows, data not shown). The number of univalents was higher in *him-6* consistent with the stronger *him* phenotype in this mutant (Figure 3C, Table 1, Table S2).

**Table 1:** him phenotype of various strains.

genotype	% males
N2 wild-type	0.1±0.1
*mus-81*	0.2±0.2
*slx-1*	0.9±0.3
*mus-81 slx-1*	0.8±0.2
*xpf-1*	2.3±0.7
*him-6*	12.7±5.3
*xpf-1; him-6*	12.7±2.4
*mus-81; xpf-1*	3.9±2.1
*slx-1; xpf-1*	2.3±0.6
*mus-81; him-6*	14.9±1.7
*slx-1; him-6*	11.2±1.4
*mus-81 slx-1; xpf-1; him-6*	10.0±0.1

% males in viable progeny scored.

Analysis of *slx-4*, as well as *[mus-81; xpf-1]*, *[slx-1; xpf-1]*, *[mus-81; him-6]* and *[slx-1; him-6]* double mutants revealed a distinct phenotype. Many chromatin masses looked like univalents but in contrast to *spo-11*, pairs of ‘univalents’ were found associated with each other (Figure 3A, red arrows). Importantly, by analysing series of Z-stacks, we found that chromatin bridges linked those pairs. This analysis clearly documented that ‘univalent pairs’ which we refer here after as dissociated bivalents, were connected in *slx-4*, *[mus-81; xpf-1]*, *[slx-1; xpf-1]*, *[mus-81; him-6]* and *[slx-1; him-6]* double mutants (Videos S2, S3, S4, S5, S6). A careful examination of these dissociated bivalents in *[mus-81; xpf-1]* mutants using a FISH probe against the 5S ribosomal RNA locus revealed that the DNA linkages indeed connected two homologous chromosomes (Figure 3B). Scanning through multiple Z-stacks for each genotype we estimate that the average number of dissociated bivalents per oocyte was 3.82 in *[mus-81; xpf-1]*, 2.35 in *[slx-1; xpf-1]*, 1.42 in *[mus-81; him-6]* and 1.57 in *[slx-1; him-6]* double mutants (Figure 3C, Table S2). None or low levels of dissociated bivalents were observed in wild-type and single mutant worms (Figure 3C, Table S2). We note that both *[slx-1; him-6]* and *[mus-81; him-6]* have fewer dissociated bivalents than *[mus-81; xpf-1]* and *[slx-1; xpf-1]*. Nevertheless, the increased incidence of these structures in *[slx-1; him-6]* and *[mus-81; him-6]* compared to *him-6* is statistically significant (P<0.01 in both cases) (Table S2). The lower incidence of dissociated bivalents in *[slx-1; him-6]* and *[mus-81; him-6]* worms is likely due to the overall lower number of homologues that undergo meiotic recombination in *him-6* backgrounds as previously reported [20], (Figure 3C). This is consistent with our observation that the number of univalents in *him-6* single mutant and in *[slx-1; him-6]* and *[mus-81; him-6]* double mutants is comparable (Figure 3C, Table S2). In contrast, *[xpf-1; him-6]* as well as *[mus-81 slx-1]* mutants did not show increased numbers of dissociated bivalents further supporting our prior genetic analysis suggesting that XPF-1 and HIM-6, as well as MUS-81 and SLX-1 might act in two redundant pathways to process joint molecules linking homologous chromosomes (Figure 1B, 3C, Table S2).

To better visualize the linkages we also analysed diakinesis chromosomes by super-resolution structured illumination microscopy (SIM). Individual chromosomes could be observed from different angles, allowing the generation of Z-stacks aligned with the orientation of individual chromosomes. This analysis confirmed that dissociated bivalents were connected by DNA bridges (Figure 3D, 3E and Videos S8, S9, S10, S11, S12, S13). The analysis also showed that one to three such linkages occurred within each dissociated bivalent. Given that chromosomes often overlap in cytological preparations and that some bridges were at the limit of resolution, the number of linkages could not be assessed easily (Figure 3D). To further investigate chromosome morphology at diakinesis, we used HTP-3 antibodies to stain for chromosomal axes and therefore further assess bivalent maturation. In wild-type and all single mutants we predominantly observed a cruciform HTP-3 pattern indicative of chiasma formation (Figure 4, upper left panels). In addition, HTP-3 stained as single tracks on *him-6* univalent chromosomes. In contrast, in *[mus-81; xpf-1]*, *[slx-1; xpf-1]*, *[mus-81; him-6]* and *[slx-1; him-6]* double mutants the cruciform HTP-3 staining pattern was dramatically reduced and instead HTP-3 was detected along the single axes of dissociated bivalents (Figure 4, lower panels). This phenotype was even more prominent when analysed by SIM microscopy (Figure 4, upper right panel). Thus, where chromosomes are not connected by chiasmata, DNA linkages appear to provide the only physical connection.

### DNA linkages observed in double mutants largely depend on SPO-11-induced DSBs

**Figure 5:**
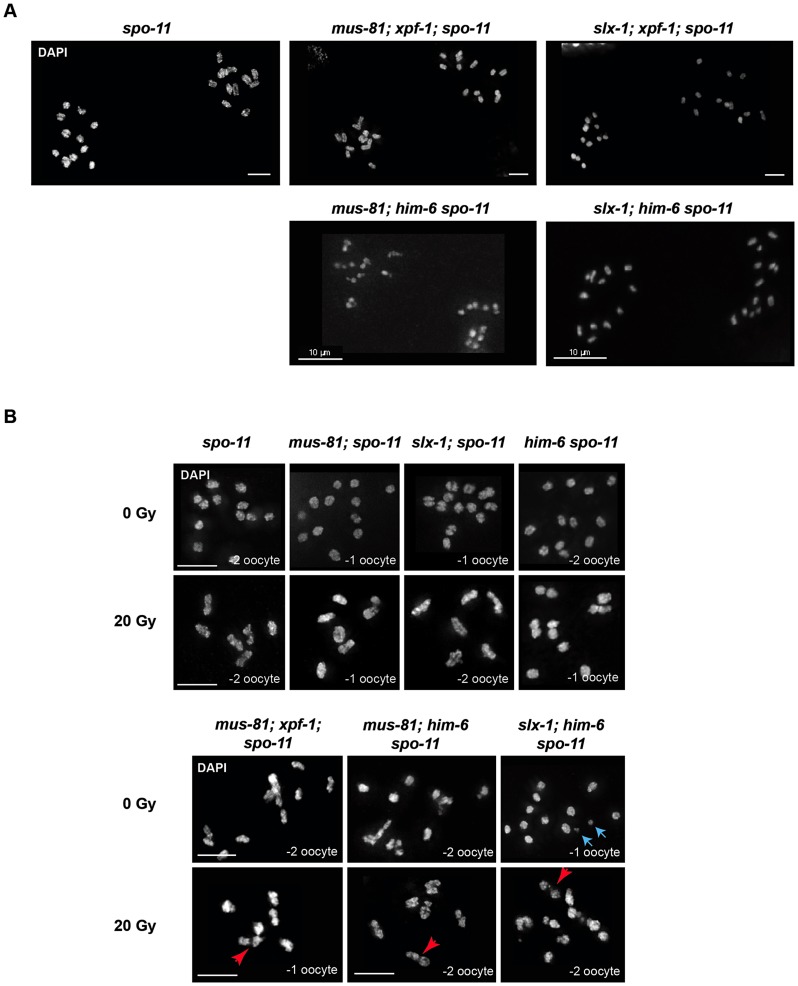
Chromosome linkages depend on SPO-11-induced meiotic double-strand breaks. (A) Images of DAPI-stained -1 and -2 oocytes (position distal to spermatheca) of the indicated genotypes. Scale bars are 4 µm unless indicated otherwise. (B) Irradiation induced double-strand breaks bypass the SPO-11 requirement. Red arrows indicate chromosome linkages. Blue arrows indicate DNA fragments, which we observed in *[slx-1; him-6 spo-11]* but not in *[slx-1; him-6]*. Scale bars are shown in white (5 µm).

Mus81, Slx1, Xpf1 and BLM homologues have been implicated in DNA repair in mitotically dividing cells in many organisms. Thus, the chromatin linkages we observed could represent unresolved DNA repair intermediates, possibly carried over from mitotic cell divisions. Alternatively, these linkages might originate from SPO-11 induced double-strand breaks, and represent unresolved meiotic RIs. To distinguish between these possibilities we combined *[mus-81; xpf-1]*, *[slx-1; xpf-1]*, *[mus-81; him-6]* and *[slx-1; him-6]* double mutants with *spo-11* to generate the respective triple mutants. Analysis of all triple mutants (except for *[mus-81; him-6 spo-11]* (see below)) indicated that chromatin linkages disappeared, and univalents could be observed, as was the case in *spo-11* single mutants (Figure 5A). We thus conclude that the linkages likely represent SPO-11-induced RIs.

It has been shown that the loss of bivalent formation in *spo-11* mutants can be rescued by IR-induced DSBs [59]. To further establish that the linkages we observed represent unrepaired meiotic RIs, we irradiated *[mus-81; xpf-1; spo-11]*, *[mus-81; him-6 spo-11]* and *[slx-1; him-6 spo-11]* mutants as well as *[mus-81; spo-11]*, *[him-6 spo-11]* and *[slx-1; spo-11]* double mutants with 20 Gy of ionizing radiation. While all un-irradiated strains that contain *spo-11* showed univalents (except for *[mus-81; him-6 spo-11]*, see below), irradiation restored bivalent formation in *spo-11* as well as *[mus-81; spo-11]*, *[slx-1; spo-11]*; and *[him-6 spo-11]* double mutants (Figure 5B). In contrast, in *[mus-81; xpf-1; spo-11]*, *[slx-1; him-6 spo-11]* and *[mus-81; him-6 spo-11]* triple mutants dissociated bivalents arose at a high frequency (Figure 5B). In summary, our data strongly suggest that the linkages detectable in the double mutant backgrounds result from unresolved meiotic RIs. We note that *[mus-81; him-6 spo-11]* triple mutants (not treated with radiation) showed a mixture of bivalents, dissociated bivalents and univalents, and occasionally also structures that looked like chromosome fusions. We speculate that these structures result from excessive DSBs occurring in pre-meiotic S-phase in *[mus-81; him-6]* double mutants that bypass the requirement for SPO-11. The depletion of Mus81 in cell lines derived from BLM patients leads to excessive genome instability is consistent with our results [60].

**Figure 6:**
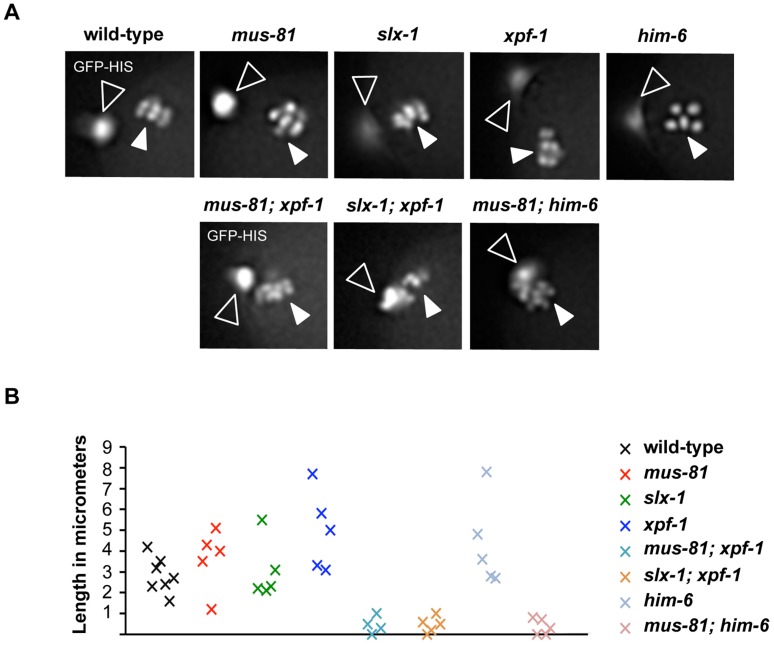
Impaired chromosome segregation during meiosis. (A) Representative images taken from time-lapse recordings of GFP-Histone H2B expressing embryos. Images were taken during metaphase of meiosis II. Open arrowheads indicate the first polar body; filled arrowheads indicate chromosomes aligned on the metaphase plate. Note five chromosomes in the *him-6* panel indicating chromosome missegregation. (B) Graph depicting the distance between the first polar body and the metaphase plate one minute prior to the onset of anaphase II. A minimum of five embryos were analysed for each genotype.

In *C. elegans* only one of several SPO-11 generated DSBs engages in CO formation between homologues [6], [59], [61], [62]. Inter-homologue RIs can also be resolved as gene conversion events. Defects in the processing of the respective intermediates might lead to the dissociated bivalent phenotype we observe. In addition it is also known that sister chromatids can be used to repair SPO-11 induced DSBs [63], [64]. Thus, to further establish if the linkages we observed indeed occur between two homologues, we examined the dynamics of meiotic chromosomes through the two meiotic divisions and also the first ensuing zygotic mitotic cell cycle. We reasoned that anaphase of meiosis I might be affected if a DNA linkage remains present between two homologues. To assess meiotic chromosome dynamics by live imaging we used an integrated Histone H2B::GFP fusion (*his-11::GFP*). Overall, we observed that single mutants behaved like wild-type (Videos S14, S15, S16, S17, S18, Figure 6A, B): during anaphase I homologous chromosomes separate. One set of chromosomes decondenses and is extruded as the first polar body. The remaining sister chromatids separate from each other during anaphase II. One set of sister chromatids is extruded as the second polar body, while the other set decondenses and undergoes DNA replication. Maternal and paternal pronuclei then meet at the centre of the zygote and fuse before the first zygotic division ensues. In contrast to wild-type and the single mutants, in *[mus-81; xpf-1]*, *[slx-1; xpf-1]* and *[mus-81; him-6]* double mutants anaphase I chromosomes did not readily separate (Videos S19, S20, S21, Figure 6A, B). It appeared as if one set of chromosomes dragged the other set of chromosomes, which decondensed, and formed the first polar body. Chromatin bridges were not always visible, likely because they were stretched and/or imaging during time-lapse microscopy was focused on a single plane. However, the first polar body remained in close vicinity to chromosomes undergoing the second meiotic division (Figure 6A, B). Only one set of anaphase II chromosomes eventually separated from the chromatin mass of the polar body. These chromosomes decondensed and engaged in the first zygotic division (Videos S19, S20, S21). During this division a modest level of chromatin bridge formation was visible. Taken together, our results suggest a defect in the segregation of homologous chromosomes during the first meiotic division, consistent with defects occurring in the resolution of inter-homologue recombination intermediates.

### Abrogation of XPF-1-HIM-6 and SLX-1-MUS-81 pathways leads to a reduction but not the elimination of CO recombination

**Figure 7:**
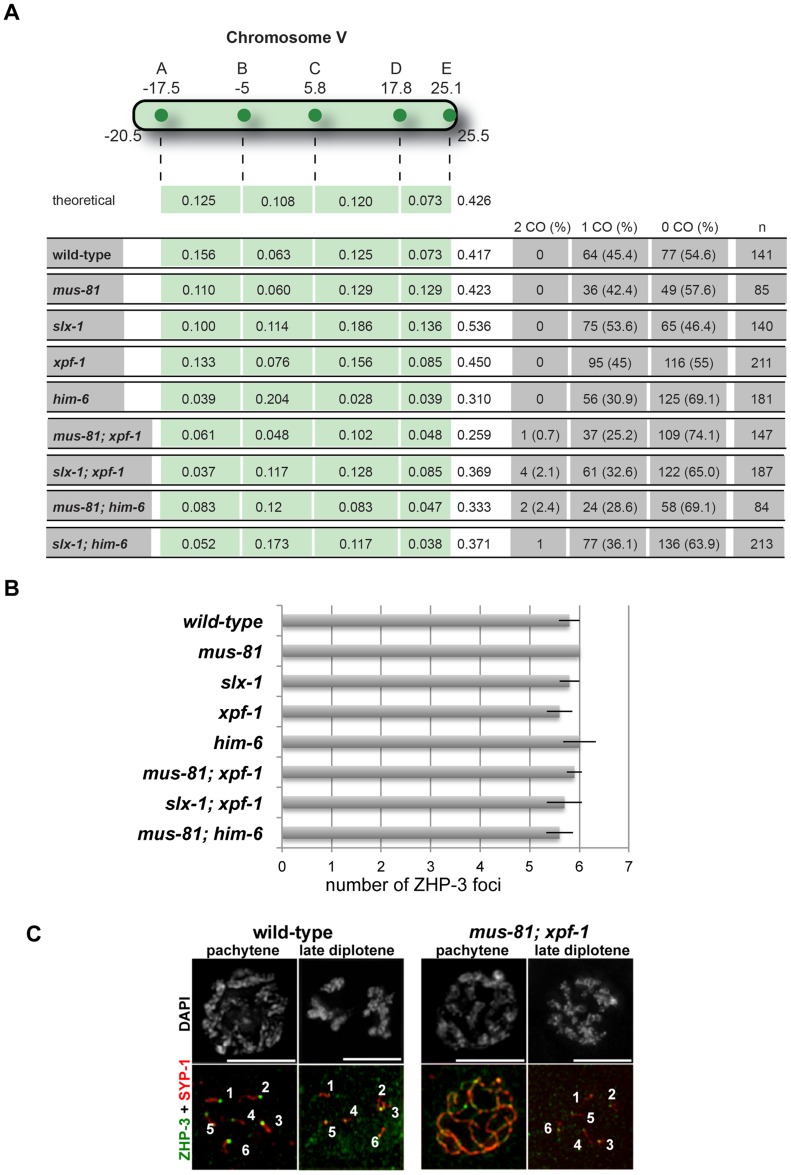
Despite showing reduced crossover recombination frequencies, wild-type numbers of ZHP-3 foci are detected in *[mus-81; xpf-1]* and *[slx-1; xpf-1]* double mutants. (A) Measurements of CO recombination frequency and distribution between five snip-SNPs, highlighted as A to E, was scored on Chromosome V. The genetic map position of the SNP used is indicated. n is the number of cross-progeny scored. The frequency of 2 COs, 1 CO or 0 CO per chromosome is indicated in absolute numbers and as percentage (in brackets). (B) Quantification of α-ZHP-3 foci in pachytene/diplotene nuclei (a minimum of 8 nuclei was scored per genotype). (C) Representative images of SYP-1 (red), ZHP-3 (green) stainings.

We next wished to directly test if meiotic recombination was reduced in *[mus-81; xpf-1]*, *[slx-1; xpf-1]*, *[mus-81; him-6]* and *[slx-1; him-6]* double mutants as predicted from our genetic and cytological data. CO frequency and distribution can be investigated by meiotic recombination mapping [10], [65], [66]. This procedure is facilitated by using multiple SNP markers along an entire chromosome that differ between the wild-type N2 ‘Bristol’ strain and the polymorphic CB4856 ‘Hawaii’ strain. We therefore generated the respective single and double mutants with chromosome V being heterozygous for Hawaii and N2. To score for recombination frequency and distribution we employed five single nucleotide polymorphisms (snip-SNPs), detectable by a change in a restriction enzyme recognition site, which together cover 92% of chromosome V (see Material and Methods, Figure 7A). *slx-1, mus-81* and *xpf-1* single mutants did not show an altered CO recombination frequency as compared to wild-type (Figure 7A). Moreover, *him-6* showed a reduced recombination rate as previously reported [21] (Figure 7A). Consistent with our idea of there being two redundant pathways for resolution comprising XPF-1-HIM-6 and MUS-81-SLX-1, we observed markedly reduced recombination frequencies in *[mus-81; xpf-1]* and *[slx-1; xpf-1]* double mutants when compared to the respective single mutants (P<0.05) (Figure 7A). Moreover, a small, but consistent number of double COs was detectable indicating that CO interference might be slightly impaired in double mutants (Figure 7A). Analysis of *[mus-81 him-6]* and *[slx-1; him-6]* double mutants did not reveal a statistically significant reduction in recombination frequency when compared to *him-6*. It is possible that a further reduction of *him-6* CO frequency by *mus-81* and *slx-1* is masked by the high frequency of chromosome V being present as univalents (Figure 3C, [30]). In summary, we observe a reduced frequency of CO recombination in *[mus-81; xpf-1]* and *[slx-1; xpf-1]* to a level comparable to the reduction described for *slx-4* [42]. Consistent with the frequency of the dissociated bivalent phenotype, as well as with the survival rates of the double mutants, we postulate that the reduction but not abolishment of CO formation indicates a reduced frequency in meiotic RIs resolution, likely HJs.

### Evidence for CO specification but not CO completion being important for the differentiation of meiotic chromosomes into distinct CO distal and CO proximal domains

The localisation of MSH-5, ZHP-3, and COSA-1 into distinct foci is interdependent and has been correlated with CO designation [61]. In wild-type germlines, ZHP-3 staining initially occurs along chromosome axes and in early diplotene congresses into six foci, one for each pair of homologues [67], [68]. In all single and double mutants analysed ∼six distinct foci eventually formed (Figure 7B,C). However, we observed a delay in the retraction of ZHP-3 from a thread-like pattern colocalizing with SYP-1 (green and red lines respectively, Videos S22, S23, S24) to six robust foci in *slx-1*, *xpf-1* and *him-6* single mutants (data not shown) as well as in double mutant germlines (Figure 7C, Videos S22, S3, S24). Similar observations had been previously reported for the *slx-4* mutant [42]. This analysis together with the reduced CO frequency observed in *slx-4* [42], *[mus-81; xpf-1]* and *[slx-1; xpf-1]* further substantiates the possibility that ZHP-3 marks a stage related to a CO precursor as opposed to a mature CO.

**Figure 8:**
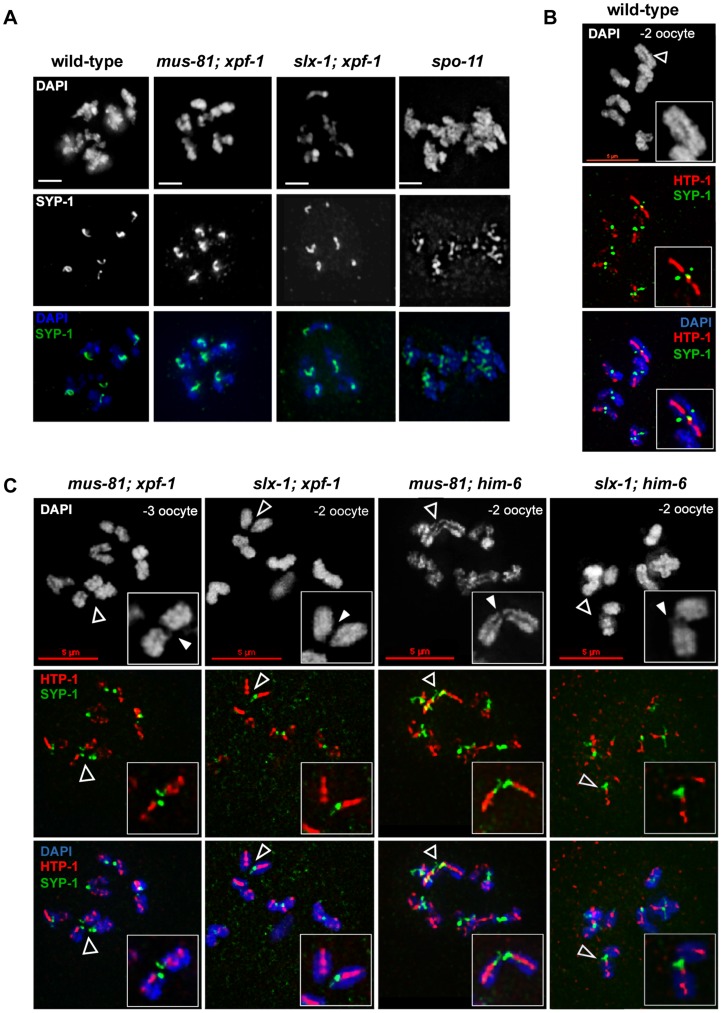
CO initiation but not CO completion is required for the reciprocal localization of HTP-1/2 and SYP-1 in diakinesis. (A) SYP-1 staining of representative diplotene nuclei. Scale bars are 2 µm. (B) HTP-1/2 (red) and SYP-1 (green) staining of wild-type diakinesis chromosomes. (C) The same staining of *[mus-81; xpf-1]*, *[slx-1; xpf-1]*, *[mus-81; him-6]* and *[slx-1; him-6]* double mutants nuclei. Filled arrowheads indicate DNA bridges. Open arrowheads indicate the chromosomes shown in the close ups. Scale bars are shown in red.

In *C. elegans* the establishment of an inter-homologue CO is thought to trigger extensive structural reorganisation of chromosomes during the late stages of meiotic prophase [69]–[71]. Therefore, we wished to address if this CO-induced chromosome restructuring depends on CO initiation or completion. In diplotene the SC asymmetrically disassembles along the paired homologues and SYP-1 localization becomes restricted to the region between the CO and the nearest chromosome end. At this stage SYP-1 can be detected as six robust threads (one for each homologue-pair) (see also Figure 8A). We clearly detected SYP-1 retracting to six robust threads after de-synapsis in late pachytene/diplotene in *[mus-81; xpf-1]* and *[slx-1; xpf-1]* double mutants (Figure 8A). As expected in the absence of COs (in *spo-11* mutants), SYP-1 was detected dispersed along entire chromosomes (Figure 8A). SYP-1 localization was variable in *him-6* mutant backgrounds, consistent with the occurrence of bivalents and univalents in these strains (data not shown).

During diakinesis, the chromosomal region in which SYP-1 is maintained matures into the short arm of the bivalent. On the short arm cohesion will be lost at the onset of anaphase I. SYP-1 localization is reciprocal to the pattern of the HTP-1/2 chromosome axis components [70], [72] (Figure 8B). HTP-1/2 becomes restricted to the long arm of the bivalent which will maintain cohesion during anaphase I. The differentiation of the CO distal and CO proximal arm is generally thought to require CO completion. We could detect SYP-1 as six robust threads in the mid-bivalent region and HTP-1/2 reciprocally localizing as a linear thread along the long-arm region of the bivalent in diakinesis oocytes of all single mutants. In *[mus-81; xpf-1]*, *[slx-1; xpf-1]*, *[slx-1; him-6]* and *[mus-81; him-6]* SYP-1 and HTP-1/2 distribution was similar to wild-type (see below) (Figure 8C). These results indicate that CO initiation but not completion is likely to be required for the timely establishment of this asymmetrical localization.

## Discussion

Our combined results suggest that XPF-1 and HIM-6, as well as SLX-1 and MUS-81 are each likely to act in a redundant pathway to resolve meiotic HJs. Both “resolvase activities” require the SLX-4 scaffold protein. Our hypothesis is supported by genetic and cytological evidence. Overall, *[mus-81; xpf-1]*, *[slx-1; xpf-1]*, *[mus-81; him-6]* and *[slx-1; him-6]* but not *[xpf-1; him-6]* and *[mus-81 slx-1]* double mutants show a similar reduction in viability as observed for *slx-4* and *[mus-81 slx-1; xpf-1; him-6]* quadruple mutants. In *slx-4* as well as in the double mutants with reduced viability, a large proportion of chromosomes fail to maintain a bivalent structure, and dissociated bivalents linked by chromatin bridges visible by high-resolution microscopy become apparent. Chromatin linkages have also been identified during the first mitotic division in the embryo and in diakinesis nuclei of *slx-4* mutants [42]. However, the prevalence of this phenotype in meiotic cells was likely underestimated. Importantly, we clearly show that these linkages largely depend on SPO-11, consistent with the hypothesis that XPF-1 and HIM-6 as well as SLX-1 and MUS-81 are required to resolve meiotic SPO-11 induced RIs. Using SIM microscopy we found that in many cases more than one chromatin linkage can be found between dissociated bivalents. Thus, the number of linkages between each dissociated bivalent is higher than the number of CO events indicating that the resolution of HJs leading to gene conversion and CO recombination might be affected.

**Figure 9:**
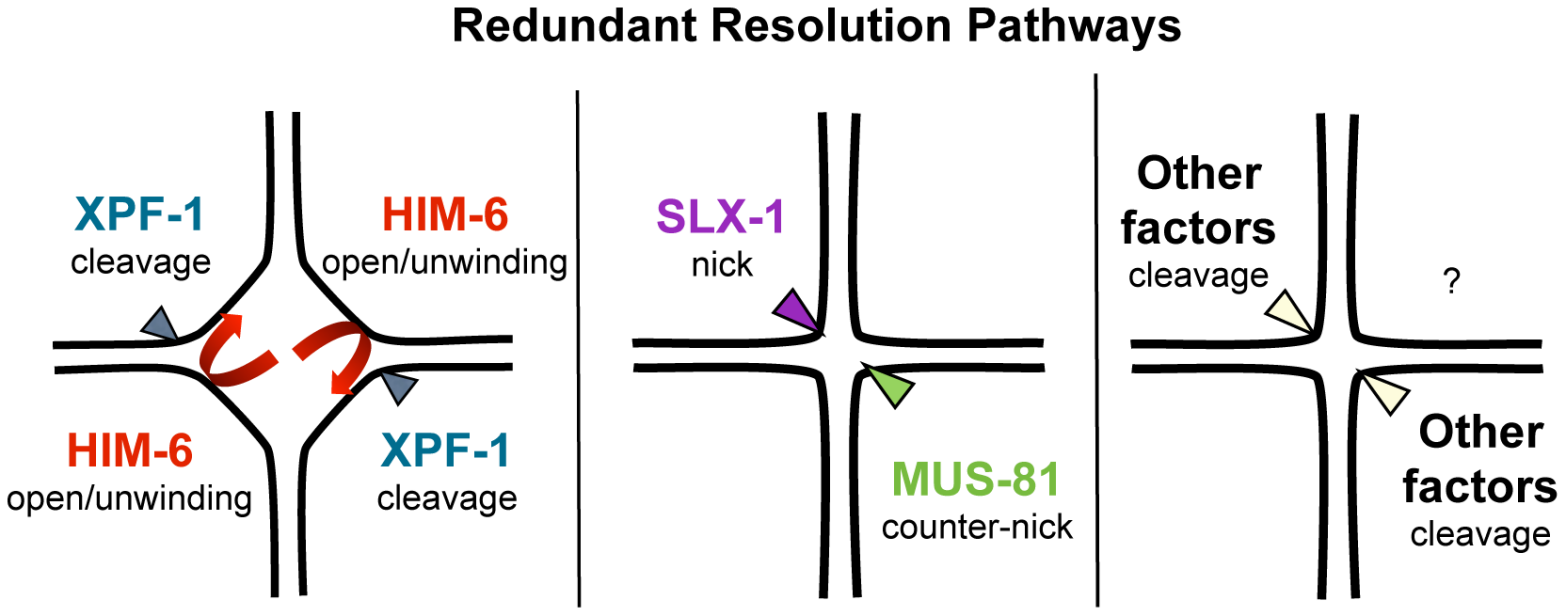
Model of redundant resolution pathways.

Our results predict that XPF-1 in conjunction with HIM-6 as well as MUS-81 in conjunction with SLX-1 might act as two distinct resolvases (Figure 9). The notion that XPF-1 and MUS-81 act in two different pathways is fully consistent with the parallel reports by O' Neil and Saito *et al.* [48], [73].

Based on the known activities of these proteins *in vitro* we speculate that these meiotic unresolved RIs are likely HJs. The study of O' Neil *et al.* further corroborates our prediction that unresolved HJs indeed accumulate in these double mutant backgrounds. By injecting an N-terminal fragment of human GEN1, the authors could rescue the dissociated bivalent phenotype in *[mus-81; xpf-1]* double mutants [73]. GEN1 symmetrically cleaves HJs, but also has some activity on FLAP structures in vitro [34].

While we report ∼15%–20% viability for *[mus-81; him-6]* and *[slx-1; him-6]* double mutants, these mutant combinations lead to 0% viability in Saito *et al* and O'Neil *et al.* [48], [73]. To maintain double mutant lines both studies used the nT1 balancer. We similarly observed 100% lethality when deriving double mutant homozygous lines from nT1 balanced *him-6* (data not shown), whereas viability is only reduced to ∼15% viability when the hT2 balancer is used. We think that the ∼15% viability is the genuine double mutant phenotype, as we were able to propagate *[mus-81; him-6]* and *[slx-1; him-6]* double homozygous mutants derived from *[mus-81/hT2 him-6]* and *[slx-1/hT2 him-6]* for several generations. Furthermore, the same phenotype was observed with a second allele of *him-6*.

How do our genetic data, which do not exclude the possibility of further proteins acting in the XPF-1-HIM-6 and the SLX-1-MUS-81 pathways, fit with the known activities of the respective enzymes? The known activities of Mus81 and Slx1 suggest a way how those two enzymes might act in conjunction to process HJs (Figure 9). Nicked HJs are a preferred substrate of Mus81. In vitro, the activity of Mus81 towards these structures by far exceeds its activity towards HJs [29]. It appears likely that Slx1 could generate such nicks. Slx1 has been shown to cleave a variety of substrates including HJs. Thus it is highly plausible that *C. elegans* SLX-1 might exert such a nickase activity at HJs [29], [47]. Given that SLX-1 and MUS-81 both bind to SLX-4 the activities of those two enzymes could be coordinated to achieve the orderly processing of HJs. Such a mechanism would be akin to classical resolvases which generally act as dimers, and which function by first conferring a nick immediately followed by a counter-nick, resulting in a complete symmetrical cleavage of a HJ [74] (Figure 9). Biochemical studies with human SLX1-SLX4 and MUS81-EME1 indeed indicate that the two nucleases act cooperatively to promote nicking and counter-nicking reactions that result in HJ resolution within the lifetime of the protein-DNA complex (HDM Wyatt, T Sarbajna, J Matos and SC West, personal communication).

*S. cerevisiae* and human Rad1/Xpf1 have been shown to cleave splayed arm, bubble and 3′ flap structures *in vitro* consistent with Xpf1's function in nucleotide excision repair and DNA single-strand annealing (reviewed in [29]). Symmetrical cleavage of a HJ substrate by Rad1 has been reported, but the interpretation of the results was disputed [75], [76]. Rad1 mutants do not have a meiotic phenotype and the ‘HJ cleavage activity’ was only observed on a ‘mobile HJ substrate’ that contains a 12 bp core of homology (X12 substrate). Such a composition allows migration of the actual junction along the core, leading to transient single-stranded structures that resemble bubble structures observed during nucleotide excision repair. Thus under certain conditions Xpf1 might introduce nicks on flexible HJ junctions without acting as a canonically resolvase that symmetrically cuts HJs such that the resulting products are readily re-ligatable. Indeed Xpf1 is discussed as a HJ resolvase/HJ processing enzyme in the fruit fly where the respective *mei-9* mutation shows a dramatically reduced level of meiotic COs [52]. Interestingly, the extent of meiotic gene conversion is not reduced in this mutant. Xpf1 might act as a HJ processing enzyme. Intriguingly, an even stronger meiotic recombination defect occurs in fly *mus312/slx4* mutants, and it has been argued that MUS312 besides MEI-9 might also need SLX1 to fully process HJs [43], [77], [78]. If *C. elegans* XPF-1 were to act as a HJ nicking enzyme, it appears possible that the HIM-6 helicase might help to unwind thermodynamically unstable HJs occurring in the context of entire chromosomes (Figure 9). It is noteworthy that electron-microscopic pictures of HJs derived from *S. pombe* meiotic cells revealed HJs that were open at their centre [25]. Thus, HIM-6 could at least transiently increase the single-strandedness of thermodynamically flexible HJ structures, thus facilitating HJs being nicked by XPF-1. At present we cannot exclude the possibility that XPF-1 and HIM-6 might act at an earlier step during meiotic recombination, acting on ‘joint molecules’ occurring prior to the formation of HJs. We know very little about HIM-6 function during *C. elegans* meiosis, but it is established that the rate of meiotic CO recombination is reduced in *him-6* mutants albeit the mechanism is not known [20]. The *S. cerevisiae* and human HIM-6 homologs Sgs1/BLM are thought to be involved in D-loop disassembly [13], [79]–[81]. Thus, the absence of HIM-6 could lead to an increased number of joint molecules, the processing of which might require MUS-81 or SLX-1.

CO recombination is reduced but not eliminated in *[mus-81; xpf-1]* and *[slx-1; xpf-1]* double mutants, while a reduction beyond the level observed in *him-6* mutants cannot be detected in *[mus-81; him-6]* and *[slx-1; him-6]*. As Saito *et al.* [48], we did not detect reduced overall CO frequencies in *slx-1*, *mus-81* and *xpf-1* when analysing chromosome V. It is possible that the reduced CO frequency on chromosome III reported by O'Neil *et al.* [73], relates to chromosome-specific differences. In addition the recombination assays employed by O'Neil *et al.*, reflect recombination rates in both male and female germ cells, while we measured recombination rates in the female germ line.

We think that these findings are consistent with our cytological observations. More dissociated bivalents are found in *[mus-81; xpf-1]* and *[slx-1; xpf-1]* while the formation of an equal number of such structures is prevented in *[mus-81; him-6]* and *[slx-1; him-6]* by the reduced rate of recombination in *him-6* which results in a reduced number of bivalents formed.

Irrespective, it is evident that further factors capable of resolving HJs must exist in the *C. elegans* germline. In principle there are two ways as to how such residual resolution might occur: 1) Redundant HJ resolving enzymes might act at the same time as SLX-4 and its associated nucleases before the onset of anaphase I. Consistent with an ‘early’ function of those redundant resolvase activities, wild-type bivalents are still present in *slx-4* mutants as well as in all double mutants. In budding yeast *mlh3*, *sgs1*, *mms4 (mus81)*, *yen1*, *slx1* quintuple mutants show a complete absence of CO products where recombination hotspot systems are employed [14], [54]. Thus EXO-1, the MutL complex and GEN-1 as well as other nucleases encoded in the *C. elegans* genome are possible candidates. 2) HJs missed by the SLX-4 associated nucleases might be processed by a backup mechanism occurring during anaphase I, or even in the second meiotic division. Such a mechanism is supported by our finding that anaphase I chromosomes appear to be linked, and only in the second meiotic division chromosomes finally seem to separate from each other. A precedent for such a mechanism is the budding yeast Yen1 resolvase [40]. This resolvase, which only acts at late stages of meiosis rescues the meiotic chromosome segregation defects associated with *mus81*. Although *C. elegans* GEN-1 is expressed in late stage oocytes (AG and Bin Wang, unpublished) it does not appear to affect meiotic HJ resolution, even when *gen-1* and *slx-4* deletions are combined. Thus other nucleases might act to ensure complete HJ resolution during anaphase I and during the subsequent second meiotic division. However, such ‘backup mechanisms’ might be rather un-physiological as they only take place in the absence of various nucleases and might fail to accurately process HJs, thus leading to a loss of genetic information. Indeed, the progeny of *[slx-1; him-6]*, *[mus-81; him-6]*, *[mus-81; xpf-1]* and *[slx-1; xpf-1]* mutants have a fluctuating viability in subsequent generations (BM and AG, unpublished). In addition, it appears possible that HJs could be torn apart by the force exerted by the spindle in the first and second meiotic divisions. The resulting DSBs would need to be repaired, likely by error-prone mechanisms.

Our quantitative analysis of *[mus-81; xpf-1]* and *[slx-1; xpf-1]* diakinesis nuclei reveals a >50% penetrance of the dissociated bivalent phenotype. If those ∼six chromosome pairs were not linked, a much higher rate of chromosome non-disjunction would be predicted. Thus, a higher incidence of males, a phenotype resulting from meiotic X chromosome non-disjunction would occur. In our hands the *him* phenotype observed in *[mus-81; xpf-1]*, *[slx-1; xpf-1]*, *[slx-1; him-6]* and *[mus-81; him-6]* double mutants is not severely enhanced compared to the phenotype observed in the respective single mutants. Chiasmata are normally needed to counteract spindle tension to ensure faithful meiotic chromosome segregation. The chromatin linkages we observed might therefore partially bypass the requirement of a chiasma to counteract tension thus explaining the relatively high rate of correct X-chromosome segregation. During diplotene and diakinesis chromosomes become highly condensed and extensive remodelling occurs to form characteristic cruciform bivalents. Chiasmata are important for the differentiation of chromosomes into short and long arms relative to the CO site [71]. The area between the CO site and the closest chromosome end differentiates into a short arm, where sister chromatid cohesion will be lost in anaphase I in order to allow for the segregation of homologous chromosomes. Cohesion loss at the onset of anaphase is mediated by AIR-2, and the direct AIR-2 dependent phosphorylation of REC-8 [82], [83]. Conversely, the long arm relative to the CO site maintains cohesion during the first meiotic division [71]. This asymmetric differentiation can be visualized by the reciprocal localization of various proteins. Synapsis proteins SYP-1, 2, 3 and 4 become restricted to the short arm beginning from early diplotene, while HTP-1/2 axis components and LAB-1 localize to the long arm [69], [70], [72], [84]–[86]. How this asymmetric differentiation is regulated remains enigmatic. The high penetrance of the dissociated bivalent phenotype, which we interpret as a failure in CO resolution allowed us to address if this asymmetry and chiasma formation depend on CO initiation or CO completion. It is clear that SPO-11 dependent DSBs are required to form chiasmata. We show that the chiasmata do not form on dissociated bivalents, thus CO completion is required for chiasma formation. In contrast, CO designation marked by six distinct ZHP-3 foci, one for each CO designated site, still occurs in *slx-*4 and *[mus-81; xpf-1]*, *[slx-1; xpf-1]*, *[slx-1; him-6]* and *[mus-81; him-6]* double mutants. Interestingly, ZHP-3 foci formation is delayed in the double mutants and importantly also in most single mutants we analysed (Figure 7 C, Videos S23, S24 and data not shown). Thus, SLX-1, XPF-1 and MUS-81 are likely to be required for the timely specification of CO designated sites. The details of these dependencies will need to be investigated in future studies. Importantly, our data suggest that CO initiation at CO designated DSB sites but not completion might be sufficient to establish the correct bivalent subdomains of meiotic chromosomes. This is evident by the predominant SYP-1 localization to the short arm of chromosomes starting from early diplotene as it occurs in wild-type. Furthermore, the reciprocal localization of SYP-1 and HTP-1/2 still occurs when linked chromosomes that lack a chiasma are analysed. We note that a faint SYP-1 signal could be occasionally observed co-localising with HTP-1/2 on the long arm in *[mus-81; him-6]* and *[mus-81; xpf-1]*, double mutants (data not shown). This result might suggest defects in the correct SC disassembly consistent with O'Neil *et al.* [73]. In future work it will be important to address how CO initiation, CO designation and the establishment of chromosomal asymmetry is regulated.

It is tempting to speculate why meiotic HJ resolution might not take advantage of symmetrically cleaving HJ resolution enzymes. Canonical HJ resolvases, which are highly active enzymes, would in principle lead to CO and gene conversion products with equal frequency. The production of CO intermediates during the repair of DSBs in mitotic cells would result in the loss of heterozygosity. With hindsight it is thus not surprising that mutants solely defective in the GEN1 HJ resolvase have no overt DSB repair defects in most organisms. In meiosis CO recombination is highly regulated and importantly restricted to a small subset of CO-designated DSB sites. Thus to maintain meiotic CO homeostasis evolution might have chosen and adapted multiple conserved nucleases to more efficiently and robustly ensure orderly meiotic CO recombination. It will be interesting to uncover further redundant nucleases required for CO recombination and to study their functional interplay.

## Materials and Methods

### *C. elegans* strains and maintenance

Strains were grown at 20°C under standard conditions [87] unless indicated otherwise. N2 Bristol was used as the wild-type strain. CB4856 Hawaii was used to generate strains for CO recombination frequency analysis. Mutant strains used in this study are listed in Table S1.

The following rearrangements and balancers were used [88]:

LG I: *hT2[bli-4(e937) let-?(q782) qIs48]*; LG III: *dpy-17(e164) unc-32(e189)/qC1[dpy-19(e1259) glp-1(q339)]*; LG IV: *nT1[qIs51], nT1[unc-?(n754) let-? qIs50]* and *mIS11 (myo2::GFP insertion near dpy-20)*

The *slx-1(tm2644)*, *ercc-1(tm1981), mus-81(tm1937) and xpf-1(tm2842)* mutants were generated and kindly provided by Shohei Mitani of the National Bioresource Project for the Nematode, Japan. All mutants are null alleles and eliminate a sizable proportion of the respective open reading frames. Details are described at the National Bioresource Project for the Nematode and on www.wormbase.org. All mutants were outcrossed for a minimum of four times to the wild-type strain to eliminate background mutations. The TG2512 *gtIs2512[Ppie-1::his-11::GFP unc-119+]* strain was generating by biolistic bombardment of pAZ132 of *unc-119(ed3)* mutants [89].

### Cytological procedures

For immunostaining of germlines, 8 to 10 (24 h post L4 stage) adults were dissected per slide. Germlines were isolated in 8 µl of 1× EBT (250 mM HEPES pH 7.4, 1.18 M NaCl, 480 mM KCl, 20 M EDTA, 5 mM EGTA, 0.1% Tween 20, 20 mM sodium azide). An equal volume of 2% formaldehyde in EBT was added to the slide carefully pipetting to allow for homogenization. Fixation was done for 5 minutes at room temperature, followed by immersion in liquid nitrogen. Coverslips were quickly removed, and post fixation was done in −20°C methanol for 1 minute, followed by permeabilization by washing 3×5 minutes in PBST (1× PBS, 0.1% Tween) at RT. Blocking was performed in PBST supplemented with 3% BSA (PBSTB) incubated for 30 min at room temperature. Primary antibodies were diluted in PBSTB and covered with a parafilm coverslip, followed by over-night incubation at 4°C in a dark humid chamber. Slides were then washed 3×10 min in PBST. Secondary antibody incubation was done at room temperature for 2 hours in PBSTB supplemented with 2 µg/µL DAPI. After washing 3×10 min in PBST, the samples were mounted in Vectashield mounting medium (Vector Laboratories, Inc.) and sealed. Primary and secondary antibodies were used at the indicated dilutions: rabbit anti-HTP-3 (1∶500) [90]; guinea pig anti-ZHP-3 (1∶250) [67]; guinea pig anti-SYP-1 (1∶500) (gift from Kentaro Nabeshima); rabbit anti-HTP-1 (1∶200) (Martinez-Perez et al. 2008). Cy3 conjugated secondary antibodies (1∶250) (Jackson Immunochemicals) and Alexa 568 labelled donkey anti-rabbit (1∶750) (Molecular Probes). For DAPI staining the final concentration used was 2 µg/µL.

### Fluorescence in situ hybridization (FISH)

PCR-amplified 5S rDNA was used as a probe for the right end of chromosome V. The 5S rDNA was labelled by PCR with cy3-dUTP (GE healthcare). Gonads were dissected as described for immunostaining, fixed in 7.4% formaldehyde, freeze cracked in liquid nitrogen and then fixed in methanol, methanol: acetone, acetone for 5 min each. FISH was performed as described previously [84], except for doing washes with PBST, and NaSCN incubations at 78°C for 10 min. Preparations were mounted in Vectashield/DAPI.

### Recordings of meiotic divisions

Embryos were dissected in isotonic growth medium for blastomeres containing 35% bovine FCS (Shelton and Bowerman, 1996). Before use, bovine FCS (heat treated for 30 min at 56°C; Invitrogen) was added. Embryos were mounted on 2% agarose pads. Vaseline patches on the slide were used to reduce the pressure of the coverslip on the embryo. Images were captured every 10 or 30 seconds using a widefield DeltaVision microscope. Exposure time was 250 milliseconds and binning used was 2×2.

### Image acquisition

Microscopy images were acquired with a Delta Vision Image restoration system (Applied Precision). Raw data obtained were analysed and deconvolved using softWoRx Suite and softWoRx Explorer software (AppliedPrecision, Issaquah, WA, USA). For SIM microscopy established protocols were followed [91], [92]. Images were acquired using a UPlanSApochromat 100× 1.4NA, oil immersion objective lens (Olympus, Center Valley, PA) and back-illuminated Cascade II 512×512 electron-multiplying charge-coupled device (EMCCD) camera (Photometrics, Tucson, AZ) on the SIM version 3 system (Applied Precision) equipped with 405-, 488-, and 593-nm solid-state lasers. Samples were illuminated by a coherent scrambled laser light source that had passed through a diffraction grating to generate the structured illumination by interference of light orders in the image plane to create a 3D sinusoidal pattern, with lateral stripes approximately 0.2 µm apart. The pattern was shifted laterally through five phases and through three angular rotations of 60° for each Z-section, separated by 0.125 µm. Exposure times were typically between 100 and 200 ms, and the power of each laser was adjusted to achieve optimal intensities of between 2,000 and 4,000 counts in a raw image of 16-bit dynamic range, at the lowest possible laser power to minimize photo bleaching. Raw images were processed and reconstructed to reveal structures with greater resolution [93] implemented on SoftWorx, ver. 6.0 (Applied Precision, Inc.). The channels were then aligned in x, y, and rotationally using predetermined shifts as measured using 100 nm TetraSpeck (Invitrogen) beads with the SoftWorx alignment tool (Applied Precision, Inc.).

### Irradiation for *SPO-11* bypass experiments

non-GFP L4s were picked from balanced lines. Worms were irradiated with 20 Gy 20 h post L4 stage using a ^137^Cs source (2.14 Gy/min, IBL 437C, CIS Bio International). Adults were dissected and DAPI stained 24 h post IR as described previously [94].

### Determining meiotic crossover recombination frequencies

Meiotic CO recombination frequencies were assayed essentially as described [42], [65], using five snip-SNPs on ChrV that differ between N2 Bristol and CB4856 Hawaii. Strains used to determine CO recombination assays were crossed into Hawaii to obtain mutant strains carrying ChrV homozygous for Hawaii DNA. Single and double mutant strains containing *slx-1* and *mus-81* were balanced with hT2. GFP positive balanced mutant males with ChrV homozygous Hawaii were then crossed with hermaphrodites of identical genotype in the N2 Bristol background to obtain mutant strains heterozygous for Hawaii. Non-GFP homozygous mutant F1 cross-progeny hermaphrodites were then crossed with males of CB5584, a *myo-2::*GFP expressing strain, which expresses high levels of green fluorescent protein in pharyngeal muscles, allowed to lay eggs for 24–48 h before removing them for genotype confirmation by PCR and *Dra*I digest. 100–200 individual F1′ GFP-positive embryos and larvae were lysed and analysed for CO recombination by PCR and *Dra*I digest. Statistical analysis of CO recombination frequencies was performed using two-tailed Fisher's exact test.

Primers used:

Chromosome V

−17.5: TTTCGGAAAATTGCGACTGT and CGCGTTTTGGAGAATTGTTT
−5: GAGATTCTAGAGAAATGGACACCC and AAAAATCGACTACACCACTTTTAGC
- 5.8: CAAATTAAATATTTCTCAAAGTTTCGG and ACATAAGCGCCATAACAAGTCG
- 17.8: GAAATTCAAATTTTTGAGAAACCC and TTCAGACCATTTTTAGAATATTCAGG
- 25.1: ACTTGACTCCTCTTTTCCATG and CTGCTAGCTCAAATACTCCC

## Supporting Information

Figure S1 Rare defects of diakinesis chromosomes in *mus-81* and *slx-1*. DAPI stained diakinesis chromosomes. Images represent projected Z-stacks obtained by deconvolution microscopy. Red arrows indicate thin DAPI stained bridges, likely between different chromosomes; the white open arrow indicates chromosome fragments. Scale bars are shown in white (5 µm). Images reflect events observed in one out of 20 oocytes analysed for *mus-81*, *slx-1*, and *[xpf-1; him-6]*.
(TIF)

Table S1 List of strains used in this study.
(DOCX)

Table S2 Tabular representation of wild-type and aberrant meiotic chromosome phenotypes shown in Figure 3C. The incidence of the dissociated bivalent phenotype between *xpf-1* and *[mus-81; xpf-1]* and *[slx-1; xpf-1]* as well as *him-6* and *[mus-81; him-6]* and *[slx-1; him-6]* is statistically significant (P<0.001). Statistical significance was determined by the two- tailed Mann-Whitney test.
(DOCX)

Video S1 Wild-type oocytes, DAPI staining.
(MOV)

Video S2 *slx-4* oocytes, DAPI staining.
(MOV)

Video S3 *[mus-81; xpf-1]* oocytes, DAPI staining.
(MOV)

Video S4 *[slx-1; xpf-1]* oocytes, DAPI staining.
(MOV)

Video S5 *[mus-81; him-6]* oocytes, DAPI staining.
(MOV)

Video S6 *[slx-1; him-6]* oocytes, DAPI staining.
(MOV)

Video S7 *spo-11* oocytes, DAPI staining.
(MOV)

Video S8 Wild-type bivalent, SIM.
(MOV)

Video S9 *[mus-81; xpf-1]* linked ‘univalent’, SIM.
(MOV)

Video S10 *[slx-1; xpf-1]* linked ‘univalent’, SIM.
(MOV)

Video S11 *[mus-81; him-6]* linked ‘univalent’, SIM.
(MOV)

Video S12 *[slx-1; him-6]* linked ‘univalent’, SIM.
(MOV)

Video S13 *spo-11* univalent, SIM.
(MOV)

Video S14 Wild-type histone::GFP, meiosis I and II.
(MOV)

Video S15 *slx-1*; histone::GFP, meiosis I and II.
(MOV)

Video S16 *mus-81*; histone::GFP, meiosis I and II.
(MOV)

Video S17 *xpf-1*; histone::GFP, meiosis I and II.
(MOV)

Video S18 *him-6*; histone::GFP, meiosis I and II.
(MOV)

Video S19 *[mus-81, xpf-1]*, histone::GFP meiosis I and II.
(MOV)

Video S20 *[slx-1; xpf-1]*, histone::GFP meiosis I and II.
(MOV)

Video S21 *[mus-81; him-6]*, histone::GFP meiosis I and II.
(MOV)

Video S22 Wild-type germline, ZHP-3 and SYP-1 staining.
(MOV)

Video S23 *[mus-81; xpf-1]*, germline, ZHP-3 and SYP-1 staining.
(MOV)

Video S24 *[slx-1; xpf-1]*, germline, ZHP-3 and SYP-1 staining.
(MOV)
