# Correction: Combinatorial Regulation of Meiotic Holliday Junction Resolution in *C. elegans* by HIM-6 (BLM) Helicase, SLX-4, and the SLX-1, MUS-81 and XPF-1 Nucleases

**DOI:** 10.1371/annotation/d8c73205-151d-4e22-89c6-3aa574037d10

**Published:** 2013-08-02

**Authors:** Ana Agostinho, Bettina Meier, Remi Sonneville, Marlène Jagut, Alexander Woglar, Julian Blow, Verena Jantsch, Anton Gartner

There were errors in the Funding section. The correct funding information is as follows: 

This work was supported by grants from the Wellcome Trust Senior Research award (090944/Z/09/Z) to AG; the BBSRC studentship to AA; CRUK Grant C303/A7399 to JB; the Austrian FWF Grant P-23638-B12 and the Berta Karlik endowment from the University of Vienna. The Scottish University Life Sciences Alliance (SULSA) and the MRC grant MR/ K015869/1 supported the use of the OMX microscope, the Wellcome Trust grant 097045/B/11/Z provided infrastructure support. The funders had no role in study design, data collection and analysis, decision to publish, or preparation of the manuscript.

