# Supplementary figures and images for: Combinatorial Regulation of Meiotic Holliday Junction Resolution in C. elegans by HIM-6 (BLM) Helicase, SLX-4, and the SLX-1, MUS-81 and XPF-1 Nucleases
Source: PLoS Genet. 2013 Jul 18;9(7):e1003591. doi: 10.1371/journal.pgen.1003591 (PMC3715425; doi:10.1371/journal.pgen.1003591)

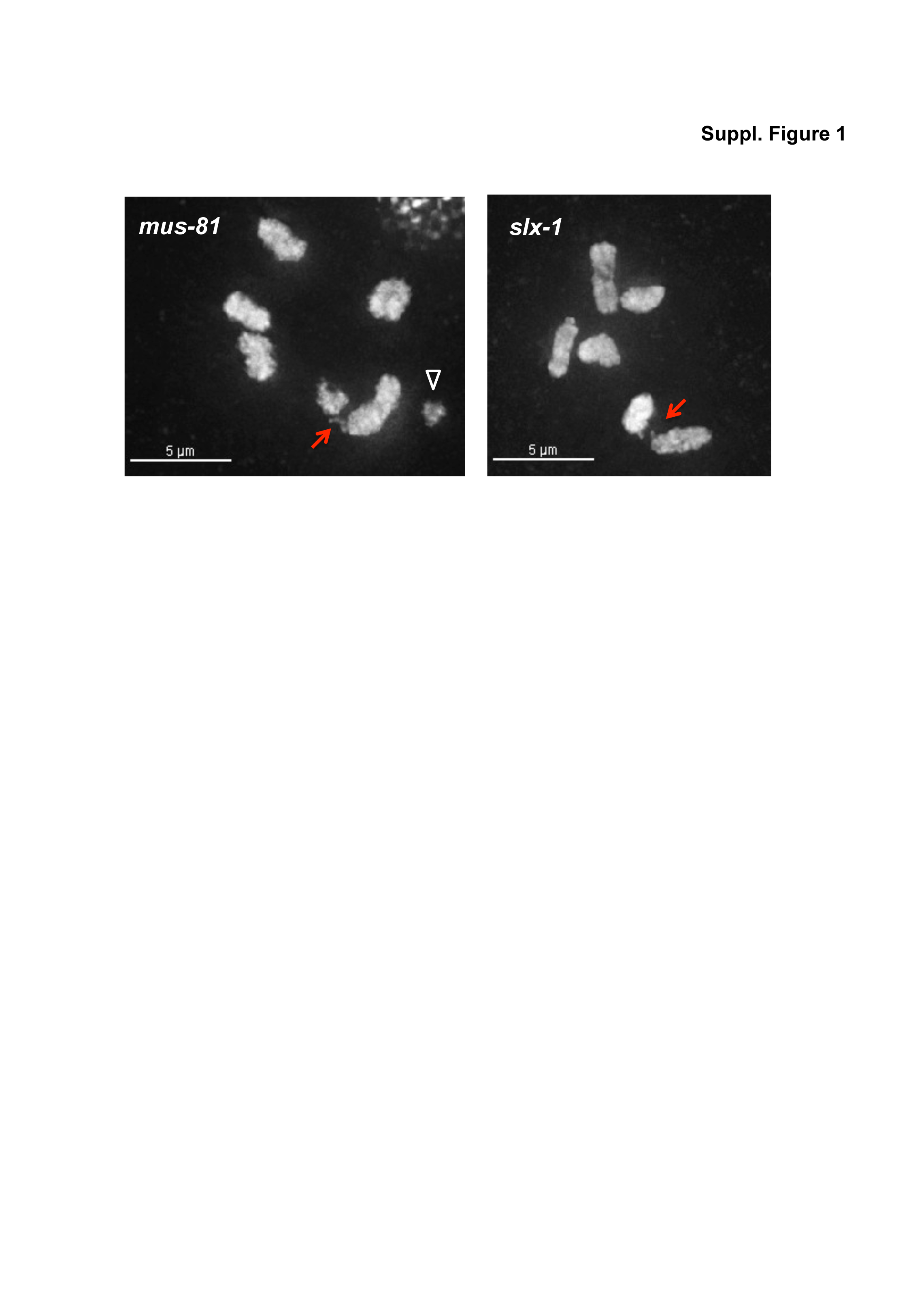

Supplement: Figure S1 — Rare defects of diakinesis chromosomes in mus-81 and slx-1. DAPI stained diakinesis chromosomes. Images represent projected Z-stacks obtained by deconvolution microscopy. Red arrows indicate thin DAPI stained bridges, likely between different chromosomes; the white open arrow indicates chromosome fragments. Scale bars are shown in white (5 µm). Images reflect events observed in one out of 20 oocytes analysed for mus-81, slx-1, and [xpf-1; him-6]. (TIF) [file pgen.1003591.s001.tif]
