# Supplementary material for: Combinatorial Regulation of Meiotic Holliday Junction Resolution in C. elegans by HIM-6 (BLM) Helicase, SLX-4, and the SLX-1, MUS-81 and XPF-1 Nucleases
Source: PLoS Genet. 2013 Jul 18;9(7):e1003591. doi: 10.1371/journal.pgen.1003591 (PMC3715425; doi:10.1371/journal.pgen.1003591)
Supplement: Table S1 — List of strains used in this study. (DOCX) [file pgen.1003591.s002.docx]

**Supplementary Table 1. List of strains used in this study.**

| TG1760 | *mus-81(tm1937) I* |
| --- | --- |
| TG1868 | *slx-1(tm2644) I* |
| TG1663 | *ercc-1(tm1981) I* |
| TG1660 | *xpf-1(tm2842) II* |
| TG1540 | *gen-1(tm2940) III* |
| TG1869 | *him-18(tm2181)/qC1 III* |
| TG1815 | *him-6(ok412) IV* |
| TG1860 | *him-6(e1104) IV* |
| AV157 | *spo-11(me44)/nT1[unc-?(n754) let-? qIs50]IV* |
| TG2512 | *gtIs2512[Ppie-1****::****his-11::GFP]* |
| TG2441 | *ercc-1(tm1981) slx-1(tm2644)/hT2 I* |
| TG2442 | *ercc-1(tm1981) mus‑81(tm1937)/hT2 I* |
| TG1890 | *mus-81(tm1937)/hT2 I; xpf-1(tm2842) II* |
| TG1974 | *mus-81(tm1937)/hT2 I; him-6(ok412) IV* |
| TG1891 | *slx-1(tm2644)/hT2 I; xpf-1(tm2842) II* |
| TG1975 | *slx-1(tm2644)/hT2 I; him-6(ok412) IV* |
| TG1878 | *mus-81(tm1937) slx-1(tm2644) I* |
| TG2455 | *xpf-1(tm2842) II; him-6(ok412) IV* |
| TG2432 | *slx-1(tm2644)/hT2 I; him-6(e1104) IV* |
| TG2434 | *mus-81(tm1937)/hT2 I; him-6(e1104) IV* |
| TG2431 | *him-18(tm2181)/qC1 III; him-6(e1104) IV* |
| TG2443 | *mus-81(tm1937) I; spo-11(me44*)/nT1 |
| TG2444 | *slx-1(tm2644) I; spo-11(me44)*/nT1 |
| TG2196 | *him-6(ok412) spo-11(me44)*/nT1 |
| TG2197 | *mus-81(tm1937) I; him-6(ok412) spo-11(me44)*/*mIS11 IV* |
| TG2214 | *slx-1(tm2644) I; him-6(ok412) spo-11(me44)*/*mIS11* IV |
| TG2215 | *mus-81(tm1937)/hT2 I; Hawaii V* |
| TG2364 | *slx-1(tm2644)/hT2 I; Hawaii V* |
| TG2445 | *xpf-1(tm2842) II; Hawaii V* |
| TG2366 | *him-6(ok412) IV; Hawaii V* |
| TG2446 | *mus-81(tm1937)/hT2 I; xpf-1(tm2842) II; Hawaii V* |
| TG2216 | *mus-81(tm1937)/hT2 I; him-6(ok412) IV; Hawaii V* |
| TG2447 | *slx-1(tm2644)/hT2 I; xpf-1(tm2842) II; Hawaii V* |
| TG2365 | *slx-1(tm2644)/hT2 I; him-6(ok412) IV; Hawaii V* |
| TG2449 | *mus-81(tm1937) I; unc-119(ed3)?III; gtIs2512[Ppie-1****::****his-11::GFP]* |
| TG2450 | *slx-1(tm2644) I; unc-119(ed3)?III; gtIs2512[Ppie-1****::****his-11::GFP]* |
| TG2451 | *xpf-1(tm2842) II; unc-119(ed3)?III; gtIs2512[Ppie-1****::****his-11::GFP]* |
| TG2429 | *unc-119(ed3)?III; him-6(ok412) IV; gtIs2512[Ppie-1****::****his-11::GFP]* |
| TG2452 | *mus-81(tm1937)/hT2 I; xpf-1(tm2842) II; unc-119(ed3)?III; gtIs2512[Ppie-1****::****his-11::GFP]* |
| TG2430 | *mus-81(tm1937)/hT2 I; unc-119(ed3)?III; him-6(ok412) IV; gtIs2512[Ppie-1****::****his-11::GFP]* |
| TG2454 | *slx-1(tm2644)/hT2 I; xpf-1(tm2842) II; unc-119(ed3)?III; gtIs2512[Ppie-1****::****his-11::GFP]* |
| TG2448 | *mus-81(tm1937) slx-1(tm2644)/hT2 I ; xpf-1(tm2842) II* |
| TG2456 | *mus-81(tm1937) slx-1(tm2644)/hT2 I ; xpf-1(tm2842) II; him-6(ok412) IV* |
| TG1757 | *mus-81(tm1937) I; gen-1(tm2940) III* |
| TG2457 | *slx-1(tm2644) I; gen-1(tm2940) III* |
| TG2458 | *mus-81(tm1937) slx-1(tm2644) I; gen-1(tm2940) III* |
| TG2460 | *gen-1(tm2940) III; him-6(ok412) IV* |
| TG2461 | *xpf-1(tm2842) II; gen-1(tm2940) III* |
| TG1870 | *gen-1(tm2940) him-18(tm2181)/qC1 III* |
| TG1871 | *mus-81(tm1937) I; him-18(tm2181)/qC1 III* |
| TG2708 | *mus-81(tm1937)I; xpf-1(tm2842)II; spo-11(me44)*/nT1 |
| TG1872 | *mus-81(tm1937) I; gen-1(tm2940) him-18(tm2181)/qC1 III* |
| CB5584 | *mIS12 II*, described in wormbase |
| PD4792 | *mIS11 VI*, described in wormbase |
