# Supplementary material for: Combinatorial Regulation of Meiotic Holliday Junction Resolution in C. elegans by HIM-6 (BLM) Helicase, SLX-4, and the SLX-1, MUS-81 and XPF-1 Nucleases
Source: PLoS Genet. 2013 Jul 18;9(7):e1003591. doi: 10.1371/journal.pgen.1003591 (PMC3715425; doi:10.1371/journal.pgen.1003591)
Supplement: Table S2 — Tabular representation of wild-type and aberrant meiotic chromosome phenotypes shown in Figure 3C. The incidence of the dissociated bivalent phenotype between xpf-1 and [mus-81; xpf-1] and [slx-1; xpf-1] as well as him-6 and [mus-81; him-6] and [slx-1; him-6] is statistically significant (P<0.001). Statistical significance was determined by the two- tailed Mann-Whitney test. (DOCX) [file pgen.1003591.s003.docx]

**Supplementary Table 2. Tabular representation of wild-type and aberrant meiotic chromosome phenotypes shown in Figure 3C.**

|  | **% of bivalents** | **% of dissociated bivalents** | **% of univalent pairs** | **% of n/d** |
| --- | --- | --- | --- | --- |
| wild-type | 98.15 | 1.85 | 0 | 0 |
| *slx-1* | 90.83 | 0 | 1.67 | 7.5 |
| *mus-81* | 93.06 | 4.17 | 0 | 2.78 |
| *xpf-1* | 78.06 | 7.10 | 8.39 | 6.45 |
| *mus-81; xpf-1* | 5.34 | 64.12 | 9.16 | 21.37 |
| *slx-1; xpf-1* | 33.67 | 40.82 | 13.27 | 12.24 |
| *him-6* | 53.82 | 4.83 | 32.53 | 8.84 |
| *mus-81; him-6* | 35.86 | 23.45 | 35.17 | 5.52 |
| *slx-1; him-6* | 44.05 | 26.20 | 22.62 | 7.14 |
| *xpf-1; him-6* | 55.36 | 1.76 | 33.93 | 7.93 |
| *slx-4* | 22.92 | 54.17 | 8.33 | 14.58 |
